# Genetic Variants Associated With Human Eye Size Are Distinct From Those Conferring Susceptibility to Myopia

**DOI:** 10.1167/iovs.62.13.24

**Published:** 2021-10-26

**Authors:** Denis Plotnikov, Jiangtian Cui, Rosie Clark, Juho Wedenoja, Olavi Pärssinen, J. Willem L. Tideman, Jost B. Jonas, Yaxing Wang, Igor Rudan, Terri L. Young, David A. Mackey, Louise Terry, Cathy Williams, Jeremy A. Guggenheim, for the UK Biobank Eye and Vision Consortium and the CREAM Consortium

**Affiliations:** 1 School of Optometry and Vision Sciences, Cardiff University, Cardiff, United Kingdom; 2 Central Research Laboratory, Kazan State Medical University, Kazan, Russia; 3 Department of Ophthalmology, University of Helsinki and Helsinki University Hospital, Helsinki, Finland; 4 Department of Public Health, University of Helsinki, Helsinki, Finland; 5 Gerontology Research Center and Faculty of Sport and Health Sciences, University of Jyväskylä, Jyväskylä, Finland; 6 Department of Ophthalmology, Erasmus Medical Centre, Rotterdam, The Netherlands; 7 Department of Epidemiology, Erasmus Medical Centre, Rotterdam, The Netherlands; 8 Department of Ophthalmology, Medical Faculty Mannheim, Heidelberg University, Mannheim, Germany; 9 Beijing Institute of Ophthalmology, Beijing Ophthalmology and Visual Science Key Lab, Beijing Tongren Eye Center, Beijing Tongren Hospital, Capital Medical University, Beijing, China; 10 Institute of Molecular and Clinical Ophthalmology, Basel, Switzerland; 11 Centre for Global Health and WHO Collaborating Centre, University of Edinburgh, United Kingdom; 12 Department of Ophthalmology and Visual Sciences, University of Wisconsin–Madison, Madison, Wisconsin, United States; 13 Centre for Ophthalmology and Visual Science, University of Western Australia, Lions Eye Institute, Perth, Australia; 14 Centre for Academic Child Health, Population Health Sciences, Bristol Medical School, University of Bristol, Bristol, United Kingdom

**Keywords:** refractive error, myopia, eye size, genetic correlation, UK Biobank

## Abstract

**Purpose:**

Emmetropization requires coordinated scaling of the major ocular components, corneal curvature and axial length. This coordination is achieved in part through a shared set of genetic variants that regulate eye size. Poorly coordinated scaling of corneal curvature and axial length results in refractive error. We tested the hypothesis that genetic variants regulating eye size in emmetropic eyes are distinct from those conferring susceptibility to refractive error.

**Methods:**

A genome-wide association study (GWAS) for corneal curvature in 22,180 adult emmetropic individuals was performed as a proxy for a GWAS for eye size. A polygenic score created using lead GWAS variants was tested for association with corneal curvature and axial length in an independent sample: 437 classified as emmetropic and 637 as ametropic. The genetic correlation between eye size and refractive error was calculated using linkage disequilibrium score regression for approximately 1 million genetic variants.

**Results:**

The GWAS for corneal curvature in emmetropes identified 32 independent genetic variants (*P* < 5.0e-08). A polygenic score created using these 32 genetic markers explained 3.5% (*P* < 0.001) and 2.0% (*P* = 0.001) of the variance in corneal curvature and axial length, respectively, in the independent sample of emmetropic individuals but was not predictive of these traits in ametropic individuals. The genetic correlation between eye size and refractive error was close to zero (*r_g_* = 0.00; SE = 0.06; *P* = 0.95).

**Conclusions:**

These results support the hypothesis that genetic variants regulating eye size in emmetropic eyes do not overlap with those conferring susceptibility to myopia. This suggests that distinct biological pathways regulate normal eye growth and myopia development.

In the United States and Europe, the prevalence of myopia is 30% to 50%, whereas in parts of East Asia myopia currently affects the majority of young adults.^1^ Not only do individuals with myopia = have the inconvenience of requiring spectacles, contact lenses, or refractive surgery to see clearly in the distance, but they also are at an increased risk of visual impairment and blindness from causes such as retinal detachment, myopic macular degeneration, cataract, and glaucoma.^2^^,^^3^

Myopia generally results from excessive axial growth of the eye during childhood, leading to a mismatch between the optical focal length of the eye and the axial length of the eye. This structural relationship between excessive axial eye length and refractive error gives rise to the rule of thumb that an axial elongation of 1 mm causes a myopic shift of approximately –2.70 diopters (D).^4^ The axial elongation associated with myopia development underlies the linear relationship between axial length and refractive error observed in individuals with axial ametropia, except for a kink in the region of emmetropia. By definition, however, no such linear relationship between axial length and refractive error exists in individuals who are emmetropic (this distinction between ametropic and emmetropic individuals can be seen in Fig. 1B). The lack of a strong relationship between axial length and refractive error in emmetropic eyes is a consequence of emmetropization, which is defined as a narrowing of the refractive error distribution, centered in the low hyperopic range, that is mediated in part by active, visually guided feedback.^5^ It is noteworthy that emmetropic eyes vary widely in size (Figs. 1A–1C); for example, axial length in the emmetropic eyes of the 15-year-olds shown in Figure 1A varied across the range of 21 to 25 mm. The kink in the axial length versus refractive error relationship is also a consequence of emmetropization; eye elongation that leads to myopia typically begins in eyes that are initially emmetropic (or that have a low level of hyperopia), and these eyes can start out being large or small. The visually guided feedback component of the emmetropization mechanism fine-tunes the rate of axial eye growth to carefully match the optical focal length of the growing eye.^6^ However, emmetropization also relies heavily on the coordinating scaling of the two major structural tissues of the eye, the cornea and sclera, to regulate overall eye size.^7^^,^^8^ Research involving young chicks, which are known to emmetropize just like humans, first demonstrated that the same genetic variants were largely responsible for regulating the radius of corneal curvature and axial eye length in this species, as evidenced by the very high genetic correlation between corneal curvature and axial length.^9^^,^^10^ Subsequent research in humans and mice indicated the general nature of this coordinated system of the eye growth, in which the genetic variants that control the rate of axial elongation during juvenile development also control the rate of flattening of corneal curvature.^11^^,^^12^ As illustrated in Figure 2, genetic correlations have desirable properties with regard to confirming or refuting causal genetic relationships between traits.

**Figure 1.**
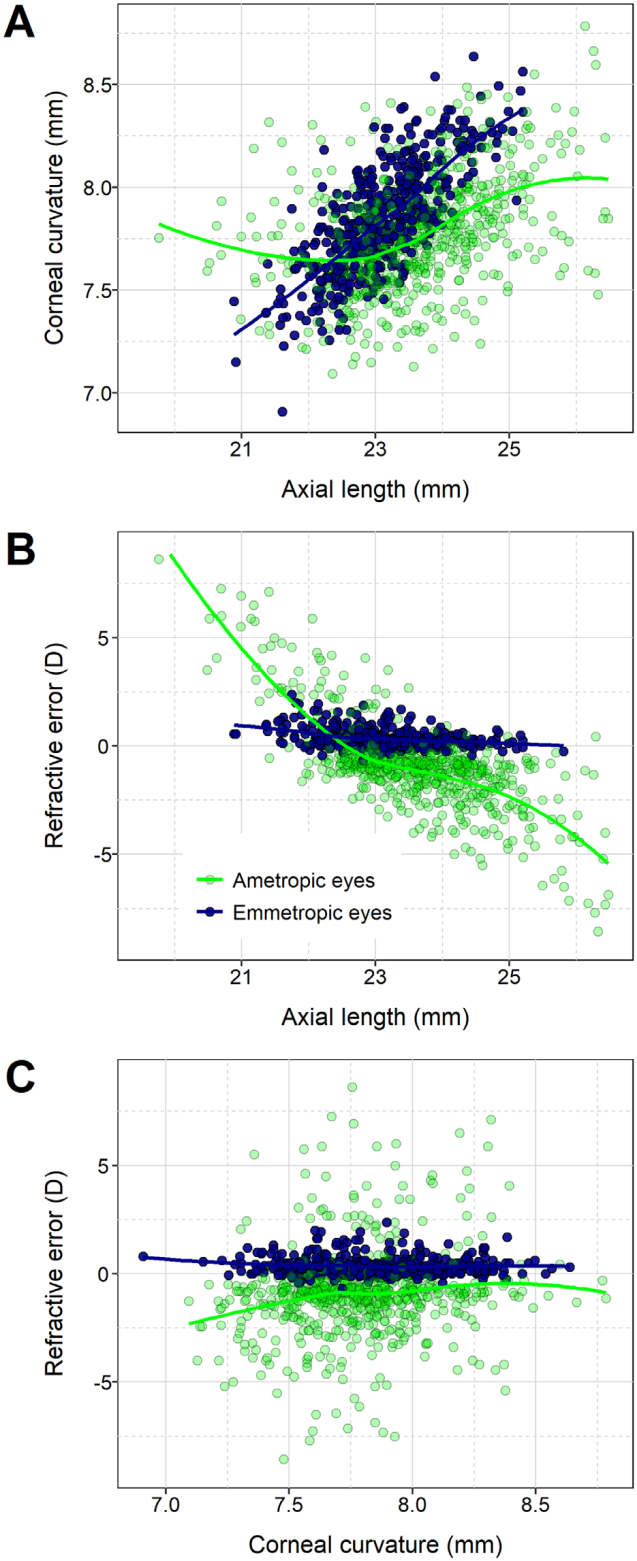
Relationships among corneal curvature, axial length, and refractive error in an emmetropes sample and an ametropes sample from the ALSPAC study (15 years old). Data are from the emmetropic eyes of 437 individuals in the emmetropes sample and the ametropic eyes of 637 individuals in the ametropes sample (the criteria for defining eyes and participants as emmetropic and ametropic are shown in Table 2). Curves were fitted using LOESS smoothing.

**Figure 2.**
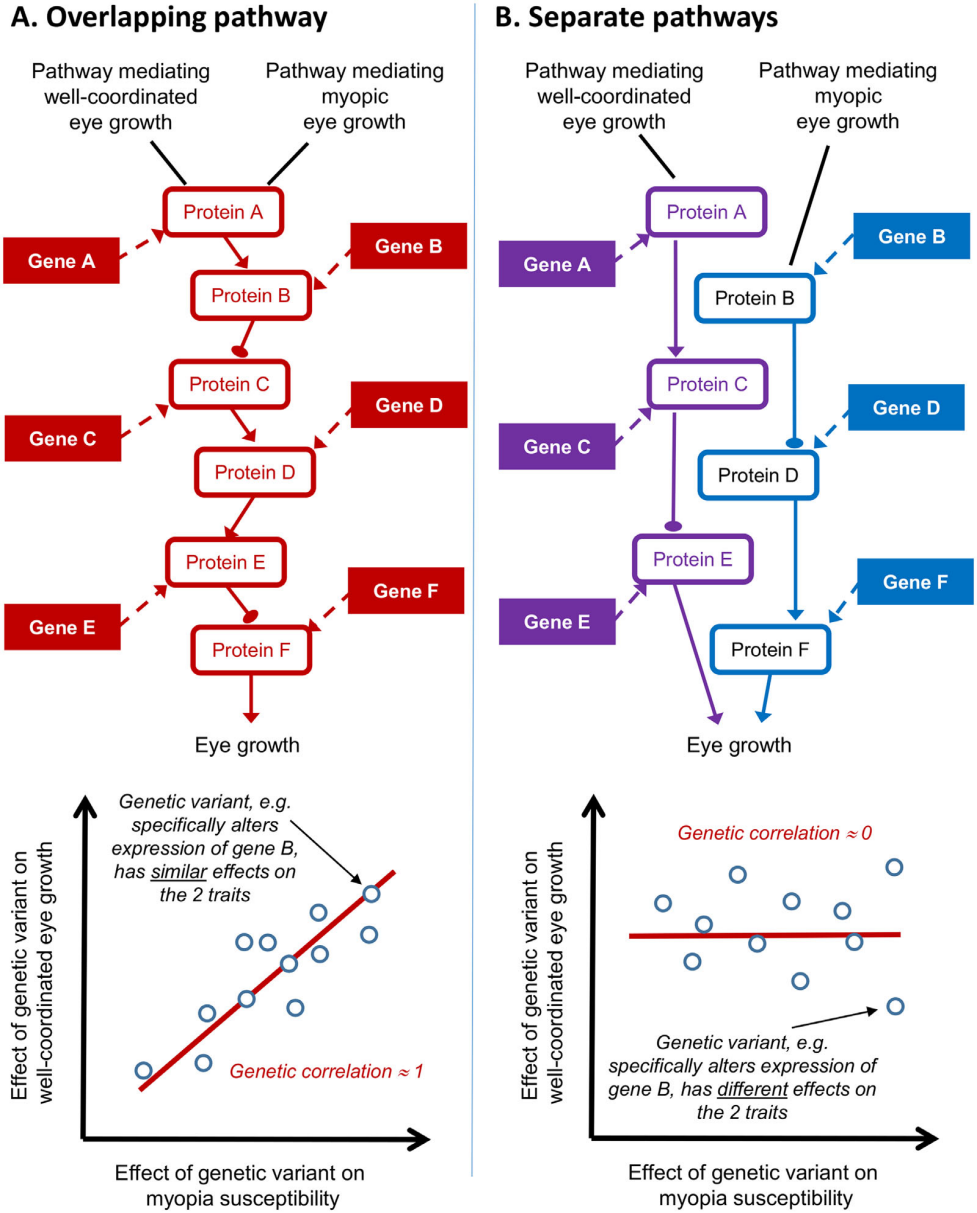
Genetic correlations can be used to draw causal inferences about the shared genetic contribution to pairs of traits. (A) Single pathway involved in both well-coordinated eye growth and myopic eye growth. (B) Two separate pathways, one that mediates well-coordinated eye growth and the other that mediates myopic eye growth. Genetic correlation analysis can distinguish whether the pathways mediating well-coordinated eye growth and myopic eye growth are overlapping or separate.

In a chick model of experimentally induced myopia, the genetic variants regulating eye size in eyes with normal visual experience were found to be distinct from the genetic variants conferring susceptibility to myopia induced by altered visual experience.^10^ Although a handful of studies in humans have each identified one or a few gene variants that fit this pattern (i.e., being associated with eye size but not associated with refractive error), the generality of this distinction has never been tested in humans.^13^^–^^16^ Here, we perform the first genome-wide association study for eye size in humans and use the results to test the hypothesis that the genetic variants controlling normal eye size do not overlap with those associated with susceptibility to refractive error.

## Methods

### Study Cohorts, Ophthalmic Assessment, and Genotyping

#### UK Biobank

The UK Biobank is a longitudinal study of the health and well-being of approximately half a million UK residents 40 to 70 years old at baseline.^17^ Ethical approval was obtained from the National Health Service National Research Ethics committee (ref. 11/NW/0382), and all participants provided informed consent. Recruitment occurred between 2006 and 2010. All participants completed a series of interviews and physical or cognitive measurements. Approximately 25% of participants underwent an ophthalmic examination introduced toward the latter stages of recruitment. This visit included a logMAR visual acuity examination at a test distance of 4 m with habitual spectacles, if worn, and non-cycloplegic autorefraction/keratometry (Tomey RC5000; Tomey GmbH Europe, Erlangen-Tennenlohe, Germany). Participants were excluded from the current analyses if they had a history of cataracts, retinal detachment, laser refractive surgery, cataract surgery, corneal graft surgery, any other eye surgery in the last 4 weeks, any eye trauma resulting in sight loss, or serious eye problems. Participants were also excluded if their hospital records indicated they had undergone cataract surgery, retinal detachment surgery, or corneal surgery. Genotyping, imputation, and genetic quality control assessment of UK Biobank participants were performed as described.^18^^,^^19^

#### Avon Longitudinal Study of Parents and Children

Ethical approval for the Avon Longitudinal Study of Parents and Children (ALSPAC) was obtained from the ALSPAC Ethics and Law Committee and the Local Research Ethics Committees. Informed consent for the use of data collected via questionnaires and clinics was obtained from participants. Pregnant women residing in Avon, UK, with expected dates of delivery between April 1, 1991, and December 31, 1992, were invited to take part in the study. When the oldest children were approximately 7 years of age, an attempt was made to bolster the initial sample with eligible cases who had failed to join the study originally. As a result, an additional 913 children were enrolled. The phases of enrollment are described in more detail in two cohort profile papers.^20^^,^^21^ The total sample size was 15,454 pregnancies. The study website contains details of all the data that are available through a fully searchable data dictionary and variable search tool (http://www.bristol.ac.uk/alspac/researchers/our-data/). Genotyping and imputation of ALSPAC participants were performed as described.^22^ ALSPAC participants were invited to attend a number of visits to an assessment center. The visit that was held when participants reached approximately 15 years of age included a vision assessment, during which refractive error was measured by non-cycloplegic autorefraction (Canon R50; Canon USA, Inc., Lake Success, NY, USA). Also, in the 50.1% of participants assessed during the final year of data collection, axial length and corneal curvature were measured by partial coherence interferometry and infrared keratometry, respectively (IOLMaster; Carl Zeiss Meditec, Jena, Germany). Of the 5501 children attending the ALSPAC assessment visit at age 15 years, 1848 had information available for refractive error, axial length, corneal curvature, height, and genome-wide genotypes, and their genetic ancestry was classified as European.^11^

### Genome-Wide Association Study for Eye Size in UK Biobank Participants

A very large sample size is required for a GWAS analysis. No such sample is available worldwide in which both the axial eye length and corneal curvature of participants have been measured. Therefore, in the current study, we took advantage of the reported coordinated genetic scaling of axial length and corneal curvature in humans^11^ by performing a GWAS for corneal curvature in emmetropes as a proxy for a GWAS for eye size. Importantly, this proxy approach is distinct from a GWAS for corneal curvature in an unselected sample of emmetropes and non-emmetropes, which will identify genetic variants associated with either eye size or refractive error.^16^

The eyes of UK Biobank participants were classified as emmetropic if the spherical (SPH) and astigmatic (CYL) refractive error lay within the range of 0.00 ≤ SPH ≤ +1.00 D and |CYL| ≤ 1.00 D, respectively, and with a habitual visual acuity < 0.2 logMAR. If both eyes were classified as emmetropic, the average corneal curvature of the two eyes was taken as the phenotype.^23^ If only one eye was classified as emmetropic, we took the corneal curvature of that eye as the phenotype (i.e., no consideration was made regarding the degree of ametropia in the fellow eye of these participants). There was a total of 22,180 individuals with at least one emmetropic eye who met the criteria for inclusion (7565 with both eyes classified as emmetropic and 14,615 with one eye classified as emmetropic). Supplementary Figure S1 outlines the selection scheme for these participants. Genetic association tests were conducted using BOLT-LMM for 6,961,902 genetic markers present on the Haplotype Reference Consortium reference panel with minor allele frequency (MAF) ≥ 0.05, IMPUTE4 INFO metric > 0.9, and per-marker and per-individual missing genotype rates < 0.02.^24^ Age, gender, genotyping array (coded as 0 or 1 for the UK BiLEVE or UK Biobank Axiom, respectively), and the first 10 ancestry principal components were included as covariates. The genetic relationship matrix for the BOLT-LMM analysis was created using a set of approximately 800,000 well-imputed variants (INFO > 0.9) with MAF > 0.005, missing rate ≤ 0.01, and an “rs” variant ID prefix that were pruned for linkage disequilibrium (LD) using the –indep-pairwise 50 5 0.1 command in PLINK 2.0.^25^ The GWAS summary statistics were filtered to remove markers with *P* < 0.01 for a test of Hardy–Weinberg equilibrium, A/T or G/C variants, and those not present in the summary statistics from the CREAM+23andMe consortium refractive error GWAS meta-analysis (see below). A set of independent markers associated at genome-wide significance (*P* < 5.0e-08) with corneal curvature in emmetropes were selected by sequentially choosing the most strongly associated marker, excluding all markers within ±500 kb of the top marker or having pairwise LD *r*^2^ > 0.2 with the top marker, and so on until there were no further markers with *P* < 5.0e-08.

### Genome-Wide Association Studies for Refractive Error, Corneal Curvature, and Body Height in UK Biobank Participants

To provide GWAS summary statistics for traits related to eye size that could be utilized in genetic correlation analyses, three additional GWAS analyses were performed: (1) a GWAS for refractive error, (2) a GWAS for corneal curvature, and (3) a GWAS for body height. These three additional GWAS analyses were performed with a sample size equal to that of the GWAS for corneal curvature in emmetropes (*n* = 22,180) (Table 1) and with the same set of 6,961,902 genetic markers. The sample of 22,180 participants for these three additional GWAS for refractive error, corneal curvature, and body height was selected at random from the set of participants with data available for refractive error, body height, visual acuity, and corneal curvature (Supplementary Fig. S1). GWAS analyses were conducted with BOLT-LMM using the same parameters and covariates as the GWAS for corneal curvature in emmetropes. Because this latter sample of 22,180 UK Biobank participants used for the three additional GWAS for refractive error, corneal curvature, and body height was drawn at random, there was an overlap (*n* = 5556) with the sample of 22,180 UK Biobank participants used for the GWAS of corneal curvature in emmetropes.

**Table 1.** Demographic Characteristics of Study Samples

	UK Biobank Participants		ALSPAC Participants			
Trait	Emmetropes Sample (*n* = 22,180)	Randomly Selected Sample^*^ (*n* = 22,180)	ALL (*n* = 1848)	Ametropes Sample (*n* = 637)	Emmetropes Sample (*n* = 437)	Other Participants (*n* = 774)
Female, % (95% CI)	56 (55–56)	54 (53–55)	54 (52–56)	56 (52–60)	54 (49–59)	52 (49–56)
Age (y), mean (95% CI)	56.74 (56.64–56.84)	57.61 (57.51–57.72)	15.46 (15.44–15.47)	15.48 (15.45–15.50)	15.44 (15.42–15.47)	15.45 (15.43–15.47)
Corneal curvature (mm), mean (95% CI)	7.88 (7.87–7.88)	7.85 (7.85–7.86)	7.82 (7.81–7.84)	7.78 (7.76–7.8)	7.84 (7.82–7.87)	7.84 (7.83–7.86)
Axial length (mm), mean (95% CI)	—	—	23.42 (23.38–23.46)	23.61 (23.53–23.70)	23.11 (23.04–23.18)	23.43 (23.38–23.48)
Refractive error (D), mean (95% CI)	+0.66 (+0.66 to +0.67)	−0.24 (−0.28 to −0.21)	−0.38 (−0.44 to −0.32)	−0.97 (−1.12 to −0.81)	+0.35 (+0.31 to +0.39)	−0.31 (−0.33 to −0.30)
Height (m), mean (95% CI)	1.690 (1.689–1.691)	1.690 (1.689–1.691)	1.69 (1.69–1.70)	1.69 (1.69–1.70)	1.69 (1.68–1.69)	1.70 (1.69–1.70)

Corneal curvature, axial length, and refractive error measurements are the average for the two eyes. Axial length was not assessed in the UK Biobank study. The age of participants refers to the age at which ocular and physical measurements were obtained. CI, confidence interval.

* A sample with the same number of participants as the Emmetropes sample was selected at random (i.e., without reference to the participants’ refractive error).

### Summary Statistics for a GWAS for Refractive Error From CREAM and 23andMe, Inc.

The CREAM Consortium and the 23andMe personal genomics company jointly published a GWAS meta-analysis for a combined phenotype of refractive error and age-of-onset of myopia.^26^ We used the summary statistics from this GWAS analysis to examine the genetic correlation between eye size and refractive error. Although a larger GWAS for refractive error has been published, we used the results from this CREAM+23andMe GWAS because UK Biobank participants were not included in the CREAM+23andMe analysis, which thus provided a completely independent sample. The original CREAM+23andMe GWAS meta-analysis was performed after genomic control (GC) correction.^26^ As GC correction can downwardly bias genetic correlations, we repeated the meta-analysis of the CREAM+23andMe GWAS summary statistics without GC correction. The final sample size of the CREAM+23andMe GWAS meta-analysis was 160,420 individuals.

### Replication of Genetic Loci Associated With Eye Size in ALSPAC Participants

A total of 1848 ALSPAC participants with genetic data available also had information available at the age of 15 years for their refractive error, axial length, corneal curvature, and body height. Among the 1848 participants, 437 were assigned to an Emmetropes sample (Table 1). Adopting the criteria shown in Table 2, these participants had at least one eye classified as emmetropic (0.00 ≤ SPH ≤ +1.00 D and |CYL| ≤ 1.00 D). For participants classified as emmetropic based on having just one eye classified as emmetropic, no consideration was made regarding the degree of ametropia in their fellow eye. A further 637 participants were assigned to an ametropes sample. These individuals had neither eye classified as emmetropic and had at least one eye classified as ametropic (where ametropia was defined as an eye with |SPH| > 1.00 D and/or |CYL| > 1.00 D). Accordingly, no participant was included in both the emmetropes sample and the ametropes sample. The trait values for refractive error, axial length, and corneal curvature were averaged for participants classified as being emmetropic in both eyes and assigned as the value in the emmetropic eye for participants emmetropic in only one eye. Trait values in ametropic eyes were assigned similarly.

**Table 2.** Definitions for Classifying Eyes and Participants as Emmetropic and Ametropic

UK Biobank Participants	ALSPAC Participants	
Emmetropic Eyes	Emmetropic Eyes	Ametropic eyes
0.00 ≤ SPH ≤ +1.00 D *and* |CYL| ≤ 1.00 D *and* < 0.2 logMAR	0.00 ≤ SPH ≤ +1.00 D *and* |CYL| ≤ 1.00 D	|SPH| > 1.00 D *or* |CYL| > 1.00 D
**Emmetropic Participants**	**Emmetropic Participants**	**Ametropic Participants**

At least one emmetropic eye	At least one emmetropic eye	Neither eye emmetropic *and* At least one ametropic eye

To test for replication of the GWAS results, each of the 32 genetic variants identified in the UK Biobank GWAS for corneal curvature in emmetropes was tested for association with each of the traits of interest (refractive error, axial length, corneal curvature, and body height) in the 1848 ALSPAC participants using linear regression. The trait of interest was considered the outcome variable. Single-nucleotide polymorphism (SNP) genotype, age in months, and sex were modeled as predictor variables. A binomial test was performed to evaluate if the variants tested for replication had a matched direction of association or achieved a *P* value below 0.1 (we adopted *P* < 0.1 for this assessment instead of the conventional *P* < 0.05 due to the limited size of the ALSPAC sample and thus limited statistical power).

The 32 genome-wide significant genetic variants identified in the GWAS for corneal curvature in emmetropes were combined to create a polygenic score.^27^^,^^28^ In order to examine if this polygenic score had the capacity to successfully predict eye size in an independent sample, a polygenic score value was calculated for each ALSPAC participant and then tested for association with either axial length or with corneal curvature. The polygenic score was derived as the weighted sum of risk alleles carried by a participant, with the regression (beta) coefficient from the UK Biobank GWAS for corneal curvature in emmetropes used as the weighting factor (as listed in Table 3). The polygenic score was computed using the –score function in PLINK 1.9.^25^ The capacity of the polygenic score to predict axial length was examined by calculating an incremental coefficient of determination (*R*^2^) for axial length, which was defined as the increase in *R*^2^ of a linear regression model with axial length as the outcome variable and including the baseline predictors age and sex, compared with a full model including the polygenic score, age, and sex. The incremental *R*^2^ was evaluated separately in the ALSPAC Emmetropes sample (*n* = 437) and the ALSPAC Ametropes sample (*n* = 637). An incremental *R*^2^ for corneal curvature, describing the capacity of the polygenic score for eye size to predict corneal curvature, was calculated analogously. Linear regression analyses were performed using R 3.5.1 (R Foundation for Statistical Computing, Vienna, Austria).

**Table 3.** Genetic Variants Associated With Corneal Curvature in Emmetropes, a Proxy for Eye Size, in UK Biobank Participants (*n* = 22,180)

Marker	CHR	POS	EA	NEA	FEA	BETA	SE	*P*	HWE-*P*	Nearest Gene	Locus Previously Associated With Corneal Curvature	Locus Previously Associated With Axial Length	Locus Previously Associated With Refractive Error
rs73175081	22	46371079	A	G	0.69	0.047	0.003	2.0e-71	0.58	*WNT7B*	Miyake^32^ Lu^14^	Miyake^32^ Lu^14^	Miyake^32^
rs9506727	13	22318853	A	G	0.64	0.024	0.003	3.6e-21	0.48	*FGF9*	Fan^16^	Fan^16^	Fan^16^
rs4074961	1	38092723	C	T	0.56	–0.020	0.002	2.8e-16	0.39	*RSPO1*	Fan^16^ Lu^14^	Cheng^15^ Fan^16^	Li^50^
rs6945610	7	47773965	T	C	0.15	0.027	0.003	3.1e-15	0.74	*PKD1L1*	—	—	—
rs56328549	2	239226553	T	G	0.91	0.032	0.004	2.3e-13	0.44	*TRAF3IP1*	Miyake^32^	Miyake^32^	Miyake^32^
rs1886772	1	1254443	G	A	0.07	0.034	0.005	1.2e-12	0.35	*INTS11*	—	—	—
rs13051496	21	47423509	C	T	0.78	0.020	0.003	8.8e-12	0.65	*COL6A1*	Fan^16^	—	—
rs1550094	2	233385396	G	A	0.30	−0.018	0.003	1.1e-11	0.74	*PRSS56*	Gal^40^ Orr^51^ Bacci^52^	Gal^40^	Fan^53^ Kiefer^36^ Tedja^26^ Pickrell^54^ Chen^55^
rs60888743	10	90051317	A	G	0.74	−0.018	0.003	2.6e-11	0.89	*RNLS*	Fan^16^	—	Fan^16^
rs35083527	12	66336692	C	T	0.80	0.020	0.003	4.2e-11	0.84	*HMGA2*	Fan^16^	Fan^16^	—
rs12503971	4	55059151	A	G	0.74	0.018	0.003	4.9e-11	0.47	*PDGFRA*	Fan^56^ Guggenheim^13^ Fan^16^	Guggenheim^13^ Fan^16^	—
rs1861630	2	217616804	T	C	0.15	0.022	0.003	1.3e-10	0.96	*LOC101928278*	—	—	—
rs7829115	8	78624559	T	C	0.32	0.017	0.003	1.3e-10	0.58	*LOC105375911*	—	—	—
rs1309572	5	64278005	A	G	0.54	−0.016	0.002	2.1e-10	0.74	*CWC27*	Fan^16^	—	Fan^16^
rs788933	4	73378390	A	G	0.43	0.015	0.002	3.7e-10	0.97	*ADAMTS3*	Fan^16^	—	Hysi^38^
rs6787409	3	135798738	T	C	0.67	0.016	0.003	4.9e-10	0.11	*PPP2R3A*	—	—	—
rs7723567	5	79344289	T	C	0.67	0.016	0.003	7.0e-10	0.61	*THBS4*	Fan^16^	—	—
rs12441130	15	74234902	T	C	0.51	0.015	0.002	1.3e-09	0.41	*LOXL1*	—	—	—
rs772383	12	77909835	A	G	0.66	−0.016	0.003	2.0e-09	0.48	*NAV3*	—	—	—
rs2733168	3	13537054	T	C	0.19	0.019	0.003	2.5e-09	0.40	*HDAC11*	Fan^16^	Fan^16^	—
rs7090376	10	102827431	T	G	0.83	−0.019	0.003	5.5e-09	0.28	*KAZALD1*	Fan^16^	—	Fan^16^ Hysi^38^
rs12517522	5	128901607	T	C	0.32	0.015	0.003	6.4e-09	0.81	*ADAMTS19*	—	Fan^16^ Koli^57^	—
rs11221633	11	129147971	T	C	0.73	0.016	0.003	1.6e-08	0.26	*ARHGAP32*	—	—	—
rs11836781	12	91817720	G	A	0.84	−0.019	0.003	1.7e-08	0.42	*LOC105369896*	—	—	—
rs4735762	8	78097322	G	A	0.66	−0.015	0.003	2.1e-08	0.68	*LOC105375907*	—	—	—
rs147287945	6	7223566	G	A	0.92	0.026	0.005	3.0e-08	0.29	*RREB1*	—	—	Hysi^38^
rs11661854	18	11240511	G	A	0.76	0.016	0.003	3.2e-08	0.61	*PIEZO2*	—	—	—
rs77757127	14	25442259	G	A	0.89	−0.021	0.004	3.5e-08	0.35	*STXBP6*	Fan^16^	—	—
rs196040	6	22084598	A	G	0.37	0.014	0.003	3.7e-08	0.93	*LINC00340*	Shah^58^	—	—
rs62048490	16	53456276	T	C	0.68	−0.014	0.003	3.7e-08	0.25	*RBL2*	—	—	—
rs1368636	8	75788406	A	G	0.91	−0.024	0.004	3.8e-08	0.83	*PI15*	—	—	—
rs3118515	9	137436314	G	A	0.68	0.014	0.003	4.1e-08	0.52	*LOC100506532*	—	—	—

CHR, chromosome; POS, genomic position (NCBI build 37); EA, effect allele; NEA, non-effect (reference) allele; FEA, frequency of effect allele; BETA, change in corneal curvature (mm) associated with each copy of the risk allele; SE, standard error of BETA; *P*, *P* value for association with corneal curvature; HWE-*P*, *P* value in test for Hardy–Weinberg equilibrium.

### Genetic Correlations

The genetic correlation is the proportion of the observed (phenotypic) correlation between two traits that can be explained by genetic variants shared between the traits. SNP heritability is the proportion of phenotype variation that can be explained by a set of genotyped genetic variants. The SNP heritability of each trait and the genetic correlation between pairs of traits were calculated using LD score regression, with the GWAS summary statistics as input.^29^^,^^30^ These calculations were performed for approximately 1,200,000 SNPs from the HapMap3 panel,^29^ according to the default settings of the LDSC^29^ package. The pairs of traits that were analyzed are listed in Table 5. For all except one of the genetic correlation analyses there was overlap between the GWAS samples used to generate the two sets of summary statistics (as described earlier in Genome-Wide Association Studies for Refractive Error, Corneal Curvature, and Body Height in UK Biobank Participants). The exception was one of the genetic correlation analyses between eye size and refractive error, for which the GWAS for refractive error was conducted in a CREAM and 23andMe Inc. sample that did not overlap with the UK Biobank. Bulik–Sullivan et al.^29^^,^^30^ reported that genetic correlations estimated using LD score regression are largely unbiased in the presence of sample overlap.

## Results

Two samples of adult participants from the UK Biobank were studied: (1) a sample of 22,180 emmetropes, and (2) a sample of 22,180 participants selected at random from those with information available for all of the traits of interest. A sample of 1848 teenagers from the ALSPAC birth cohort were studied as a replication sample. Among the ALSPAC participants, 637 individuals were classified as ametropes and 437 individuals were classified as emmetropes (leaving 774 individuals who did not meet the inclusion criteria for either the ametropes or emmetropes sample). The demographic characteristics of the participants are shown in Table 1. The criteria for classifying eyes and participants as emmetropic and ametropic are shown in Table 2.

### Genetic Variants Associated With Eye Size

A GWAS for corneal curvature in emmetropes was performed as a proxy for a GWAS for eye size. (Ideally, a GWAS for axial length, or axial length and corneal curvature combined, would have served as a better GWAS for eye size; however, a very large, genetically profiled cohort with axial length measurements does not exist yet, to our knowledge). After testing 6,961,902 genetic markers in a sample of 22,180 UK Biobank participants, a total of 32 independent genetic regions harbored markers associated with corneal curvature in emmetropes at genome-wide significance (*P* < 5.0e-08). Details of these 32 genetic variants are listed in Table 3. In support of the validity of the approach of using corneal curvature in emmetropes as a proxy for eye size, a proportion of the most strongly associated variants in the current GWAS were in genomic regions previously reported to be associated with eye size (or with both axial length and corneal curvature). For example, the most strongly associated variant we identified, rs73175081 (*P* = 2.0e-71), is an intronic variant in *WNT7B*, the gene coding for Wnt family member 7B. This variant is in strong LD with *WNT7B* variant rs10453441 (*r*^2^ = 0.77), which has previously been associated with eye size.^14^^,^^31^^,^^32^ The second most strongly associated variant, rs9506727 (*P* = 3.6e-21), lies upstream of the *FGF9* gene and is in perfect LD (*r*^2^ = 1.00) with rs9506725, previously associated with axial length, corneal curvature, and refractive error.^16^ The third most strongly associated variant, rs4074961 (*P* = 2.8e-16), is an intronic variant in *RSPO1*, which encodes R-spondin-1. This variant has also been associated with axial length, corneal curvature, and high myopia.^14^^–^^16^ Genetic variant rs12503971 (*P* = 4.9e-11) is located in the promoter region of the *PDGFRA* gene, which codes for platelet-derived growth factor receptor A. Variants in this region have previously been associated with corneal curvature (rs2114039; *r*^2^ = 0.53),^33^^,^^34^ and, indeed, this was the first genetic locus associated with eye size in humans (rs6554163; *r*^2^ = 0.84).^13^

### Replication of Genetic Associations With Eye Size in an Independent Sample (ALSPAC)

Participants from the ALSPAC cohort with information available for corneal curvature, axial length, refractive error, and body height at the age of 15 years were evaluated as a replication sample. The much smaller size of the ALSPAC sample (*n* = 1848) compared with the GWAS discovery cohort (*n* = 22,180) resulted in limited statistical power for replication. However, the direction of association (i.e., the sign of the regression coefficient) in the original GWAS matched that in the ALSPAC replication sample more often than expected by chance for both corneal curvature and axial length (Supplementary Fig. S2). Specifically, for corneal curvature 28 out of the 32 variants had the same direction of association (*P* = 9.65e-06), whereas for axial length 25 of the 32 variants had the same direction of association (*P* = 1.05e-03). Conversely, concordance in the direction of association was no higher than expected by chance for refractive error and body height. For refractive error, the direction matched for 17 of the 32 variants (*P* = 0.430). For height, the direction matched for 18 of the 32 variants (*P* = 0.298). Furthermore, the association *P* values were lower than expected by chance for corneal curvature and axial length (Supplementary Fig. S2). For example, 10 of the 32 variants achieved *P* < 0.10 for corneal curvature (*P* = 8.09e-04), and seven of the 32 variants achieved *P* < 0.10 for axial length (*P* = 3.59e-02). However, the association *P* values were not lower than expected by chance for the other two traits: 1 of the 32 variants achieved *P* < 0.10 342 for refractive error (*P* = 0.966) and 4 of the 32 variants achieved *P* < 0.10 for body height (*P* = 0.399).

As a further test of replication, we examined how much of the variance in corneal curvature and axial length could be explained by a polygenic score created as the weighted sum of the 32 genome-wide significant variants identified in the GWAS for eye size. The polygenic score for eye size explained 3.5% (*P* = 5.69e-05) of the variance in corneal curvature and 2.0% (*P* = 1.41e-03) of the variance in axial length in a sample of emmetropes from the ALSPAC cohort (Table 4). In contrast, the polygenic score was not associated with corneal curvature (*P* = 0.070) or axial length (*P* = 0.747) in a sample of ametropes from the ALSPAC cohort, nor was the polygenic score associated with refractive error or body height in either the sample of emmetropes or the sample of ametropes (Table 4). An allele score created as the unweighted sum of 32 genome-wide significant variants was not associated with any of the four traits in either the sample of emmetropes or the sample of ametropes (Supplementary Table S1). The lack of association for the allele score compared with the polygenic score suggests that the weighting of variants when calculating the polygenic score was critical to its performance, as expected from previous studies.^35^

**Table 4.** Polygenic Score for Eye Size Was Associated With Corneal Curvature and Axial Length in an Independent Cohort

	Emmetropic Sample (*n* = 437)		Ametropic Sample (*n* = 637)	
Trait	Incremental *R*^2^	*P*	Incremental *R*^2^	*P*
Corneal curvature	0.035	5.69e-05	0.003	7.04e-02
Axial length	0.020	1.41e-03	−0.001	7.47e-01
Refractive error	−0.001	5.41e-01	−0.001	4.84e-01
Height	−0.001	5.47e-01	−0.001	9.20e-01

The increase in variance explained (incremental *R*^2^) was calculated for a linear regression model with versus without a polygenic score for eye size as a predictor variable, compared with a baseline model with the predictors of age and sex. Models were fitted for 15-year-old individuals from the ALSPAC cohort who were classified as an Emmetropic sample or an ametropic sample according to the criteria in Table 2. Incremental *R*^2^ is the increase in adjusted coefficient of determination of the full model versus the baseline model. *P* values are for a test of the null hypothesis of no improvement in fit of the full model versus the baseline model.

### Genetic Correlation Between Eye Size and Refractive Error

**Figure 3.**
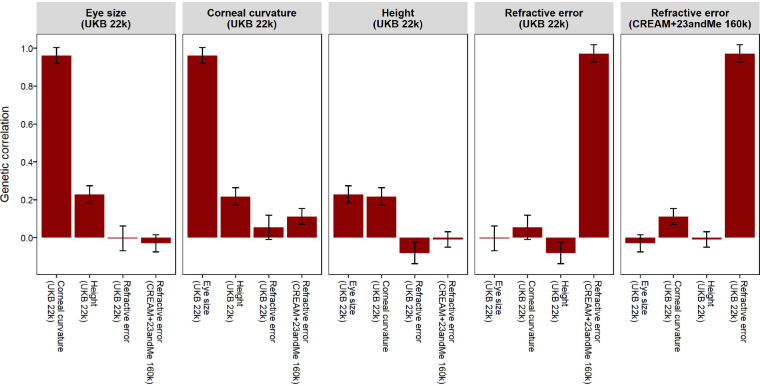
Pairwise genetic correlations among eye size, refractive error, corneal curvature, and height. Error bars represent standard errors.

Having demonstrated that the GWAS for corneal curvature in emmetropes successfully identified genetic variants associated with eye size, we tested the genetic correlation (*r_g_*) between the eye size surrogate and a series of other traits: refractive error, corneal curvature, and body height (Table 5, Fig. 3). Importantly, these genetic correlation analyses were based on a set of over 1 million genetic variants distributed across the genome, rather than just the 32 genetic variants identified as being genome-wide significantly associated with corneal curvature in emmetropes. Our key finding was that the genetic correlation between eye size and refractive error was close to zero. Specifically, *r_g_* = 0.00 ± 0.06 (*P* = 0.953) and *r_g_* = −0.03 ± 0.05 (*P* = 0.513) when the genetic correlation was computed using two separate sets of GWAS summary statistics (Table 5). In contrast, the genetic correlation between eye size and corneal curvature was very high (*r_g_* = 0.96 ± 0.04; *P* = 5.71e-119) and the genetic correlation between eye size and body height was also well above zero (*r_g_* = 0.23 ± 0.05; *P* = 3.71e-07). All of the traits considered had moderate SNP-heritability ($h_{SNP}^{2}$) values with small standard errors, suggesting that the genetic correlation calculations were well powered (Table 5). For example, $h_{SNP}^{2}$ = 0.42 ± 0.04 for eye size, $h_{SNP}^{2}$ = 0.51 ± 0.05 for body height, and $h_{SNP}^{2}$ = 0.30 ± 0.03 and $h_{SNP}^{2}$ = 0.19 ± 0.01 for refractive error in UK Biobank and CREAM+23andMe, respectively. These genetic correlation estimates were not altered appreciably when other criteria were used to define “emmetropia” when performing the GWAS for corneal curvature in emmetropes or when the GWAS for corneal curvature included body height as a covariate (Supplementary Fig. S3).

**Table 5.** Heritability and Pairwise Genetic Correlation Between Traits

Trait 1	Trait 2	Heritability of Trait 1 ($h_{SNP}^{2}$ ± SE)	Genetic Correlation (*r_g_* ± SE)	*P*
Corneal curvature (UKB; 22k)	Eye size (UKB; 22k)	0.42 ± 0.04	0.96 ± 0.04	5.71e-119
Height (UKB; 22k)	Eye size (UKB; 22k)	0.51 ± 0.05	0.23 ± 0.05	3.71e-07
Refractive error (UKB; 22k)	Eye size (UKB; 22k)	0.30 ± 0.03	0.00 ± 0.06	9.53e-01
Refractive error (CREAM+23andMe; 160k)	Eye size (UKB; 22k)	0.19 ± 0.01	−0.03 ± 0.05	5.13e-01
Eye size (UKB; 22k)	—	0.42 ± 0.04	—	—

*P* values are for a test of the null hypothesis that the genetic correlation is different from zero. UKB, UK Biobank; 22k, sample size of 22,180; 160k, sample size of 160,420.

## Discussion

GWAS analyses have discovered hundreds of genetic variants associated with refractive error and myopia,^26^^,^^36^^–^^38^ revealing that naturally occurring genetic variation impacts these conditions. Much less is known about the role of natural genetic variation in regulating eye size. In the current work, 32 genetic variants were observed to be independently associated with corneal curvature in emmetropes, and two lines of evidence suggest that these variants are associated with eye size. First, for the majority of the 32 variants, the direction of association in the original GWAS was matched in GWAS analyses for both axial length and corneal curvature in an independent sample of emmetropes. Second, a polygenic score created using the 32 variants was able to explain 2.0% of the variance in axial length and 3.5% of the variance in corneal curvature in an independent sample of emmetropic participants. Importantly, the polygenic score was not associated with axial length, corneal curvature, refractive error, or body height in a sample of ametropes, supporting its specificity for eye size in eyes with well-coordinated scaling of axial length and corneal curvature. Interestingly, 14 of the 32 genetic variants associated with corneal curvature in emmetropes in the current study were located in regions not previously linked to eye size or refractive error. The nearest genes to the lead variants in these regions were *ARHGAP32*, *INTS11*, *LOC100506532*, *LOC101928278*, *LOC105369896*, *LOC105375907*, *LOC105375911*, *LOXL1*, *NAV3*, *PI15*, *PIEZO2*, *PKD1L1*, *PPP2R3A*, and *RBL2*.

To assess whether genetic variants associated with eye size are, in general, also associated with susceptibility to refractive error, we calculated the genetic correlation between eye size and refractive error for a set of approximately 1 million SNPs distributed across the genome. This yielded a genetic correlation very close to zero, implying that there is very little genetic overlap between these two traits. This finding replicates the genetic correlation of zero between baseline (pre-treatment) eye size and susceptibility to form-deprivation myopia reported in chickens.^10^ The near-zero genetic correlation between eye size and myopia susceptibility is important to consider when interpreting the results of a GWAS for axial length or a GWAS for corneal curvature. Such a GWAS will identify a mixture of variants, some associated with refractive error and others associated with eye size, but rarely variants associated with both traits. This distinction has been recognized by some past studies, but not all.^15^^,^^16^^,^^39^ For the 32 genetic variants associated with corneal curvature in emmetropes, it was noteworthy that *FGF9* variant rs9506725 and *RSPO1* variant rs4074961 were previously found to be associated with refractive error, as well as with eye size. A prior study^16^ that specifically investigated this issue found the *RSPO1* variant to be much more strongly associated with corneal curvature and axial length than with refractive error (*P* = 1.06e-29, *P* = 2.72e-13, and *P* = 1.30e-02, respectively) but that the *FGF9* variant was strongly associated with all three traits. Taken in the context of the near-zero genetic correlation between eye size and refractive error found in the current study, which was based on an analysis of more than a million variants distributed across the genome, these variants in *FGF9* and *RSPO1* appear to be exceptions to the rule, as they exert effects on both eye size and refractive error.

Three of the candidate genes discovered in our GWAS for eye size – *PRSS56*, *ADAMTS19*, and *PIEZO2* – are of particular interest by virtue of their known or suggested role in causing nanophthalmos or microphthalmos.^40^^–^^43^ In particular, *PIEZO2* encodes piezo-type mechanosensitive ion channel component 2, a transmembrane protein that senses mechanical forces experienced by tissues and translates them into cellular signals. Thus, we suggest *PIEZO2* may act as a sensor of eye size. Genes such as *PRSS56* (and *MFRP*), for which loss of function causes nanophthalmos, have been considered potential targets for slowing the progression of myopia, because reducing their functional activity may prevent further ocular elongation.^43^^–^^46^ The current work adds to the already recognized concern that modifying the activity of these genes risks halting the normal development of the eye if initiated too early in infancy or childhood.

Strengths of this work were that it took advantage of the very large sample of individuals with emmetropia in the UK Biobank study; the use of a replication cohort (ALSPAC) with information about refractive error, axial length, corneal curvature, and body height; the standardized ocular phenotyping methods adopted by the UK Biobank and ALSPAC; and the implementation of a series of sensitivity analyses to confirm that the findings were robust. The major weakness of the current work was the need to use corneal curvature in emmetropes as a proxy for eye size rather than a dataset with information on both corneal curvature and axial length in emmetropes. Axial length was not measured in the UK Biobank, and no very large sample of emmetropes with information about both axial length and genetic data has been collected to our knowledge (for example, the CREAM Consortium GWAS for axial length study included only 12,500 participants of European ancestry, of whom only about a quarter would have been emmetropic).^15^ Ideally, instead of performing a GWAS for corneal curvature, we would have performed a GWAS jointly for corneal curvature and axial length in a large sample of emmetropes. There was evidence that our use of the proxy phenotype biased our results to be more strongly associated with corneal curvature in emmetropes than with axial length in emmetropes. For example, in the independent ALSPAC sample, the polygenic score for eye size explained 3.5% of the variance in corneal curvature compared with 2.0% of the variance in axial. Moreover, fewer of the 32 genome-wide significant variants demonstrated evidence of replication with axial length than with corneal curvature. A further limitation was that our analyses were restricted to participants of European ancestry. This restriction was necessary because genetic association studies in mixed ancestry samples are susceptible to false-positive associations resulting from population stratification.^47^ The very small phenotypic effect size typically found for individual genetic variants associated with polygenic traits necessitates the use of the largest possible sample size when seeking to discover or replicate genetic associations.^48^ Thus, in order to maximize the sample size for the current GWAS and replication analyses, we classified participants as emmetropic even if only one eye of their eyes met our criteria for emmetropia (Table 2). A disadvantage of our chosen approach is that it would have added “noise” to the GWAS, as only the phenotype value of the emmetropic eye of these participants was used in the analysis, whereas the phenotype of the fellow, non-emmetropic eye was ignored. We reasoned this disadvantage would be more than offset by the approximately threefold increase in sample size (22,180 vs. 7565) compared with classifying participants as emmetropic only if both eyes met our criteria for emmetropia. Evidence that our chosen approach of classifying participants as emmetropic did not bias the results was provided by the sensitivity analysis presented in Supplementary Figure S3b. For this sensitivity analysis, only participants classified as emmetropic in both eyes were included in the GWAS analysis and yet the resulting genetic correlations were very similar to those obtained using the original approach. A wide range of alternative criteria could be envisaged for classifying eyes and participants as emmetropic—for example, considering just the phenotype value of each participant's right eye. Despite these potential methods not having been tested exhaustively, the sensitivity analyses presented in Supplementary Figures S3a and S3b provide some reassurance regarding the robustness of our key findings to such criteria. A further limitation was that refractive error in the 15-year-old ALSPAC sample was measured without cycloplegia, which would have led to reduced accuracy in classifying eyes as emmetropic or ametropic.^49^

In summary, a GWAS analysis identified 32 genetic variants strongly associated with corneal curvature in emmetropes, 14 of which were in regions not previously associated with eye size or refractive error. In an independent group of participants, a polygenic score derived using these 32 variants was associated with eye size in emmetropic eyes that had well-coordinated scaling of corneal curvature and axial length. However, the polygenic score was not associated with eye size in ametropic eyes. More generally, our work suggests, in humans, there is little overlap of genetic variants controlling normal eye enlargement during juvenile development and variants causing susceptibility to refractive error and myopia. These findings imply that therapies for myopia that target the products of genes such as *PRSS56*, *MFRP*, and *ADAMTS19* may modify distinct biological pathways compared with those upregulated to produce myopic eye enlargement. Currently, it is unknown if this is an advantage or a disadvantage.

## Supplementary Material

Supplement 1
