# Supplementary material for: Genetic Variants Associated With Human Eye Size Are Distinct From Those Conferring Susceptibility to Myopia
Source: Invest Ophthalmol Vis Sci. 2021 Oct 26;62(13):24. doi: 10.1167/iovs.62.13.24 (PMC8556552; doi:10.1167/iovs.62.13.24)

Genetic variants associated with human eye size are distinct from those conferring susceptibility to myopia

Supplementary Information

Table S1. Association of an unweighted allele score for eye size with corneal curvature, axial length, refractive error and height. The increase in variance explained (‘incremental R^2’) was calculated for a linear regression model with vs. without an unweighted allele score for eye size as a predictor variable, compared to a baseline model with the predictors age and sex. Models were fitted for 15-year-old individuals from the ALSPAC cohort whose eyes were classified as emmetropic or ametropic.

**Trait**	**Emmetropic sample (n=437)**		**Ametropic sample (n=637)**	
	**Incremental R^2**	***P*-value**	**Incremental R^2**	***P*-value**
Corneal curvature	-0.001	4.21E-01	0.000	3.22E-01
Axial length	0.000	3.73E-01	-0.001	7.34E-01
Refractive error	0.000	2.78E-01	-0.001	6.59E-01
Height	-0.001	4.66E-01	0.000	2.57E-01

Incremental R^2 is the increase in adjusted coefficient of determination of the full model vs. the baseline model.
P-value is for a test of the null hypothesis of no improvement in fit of the full model vs. the baseline model.

Figure S1. Selection of emmetropic UK Biobank participants for the GWAS analyses.

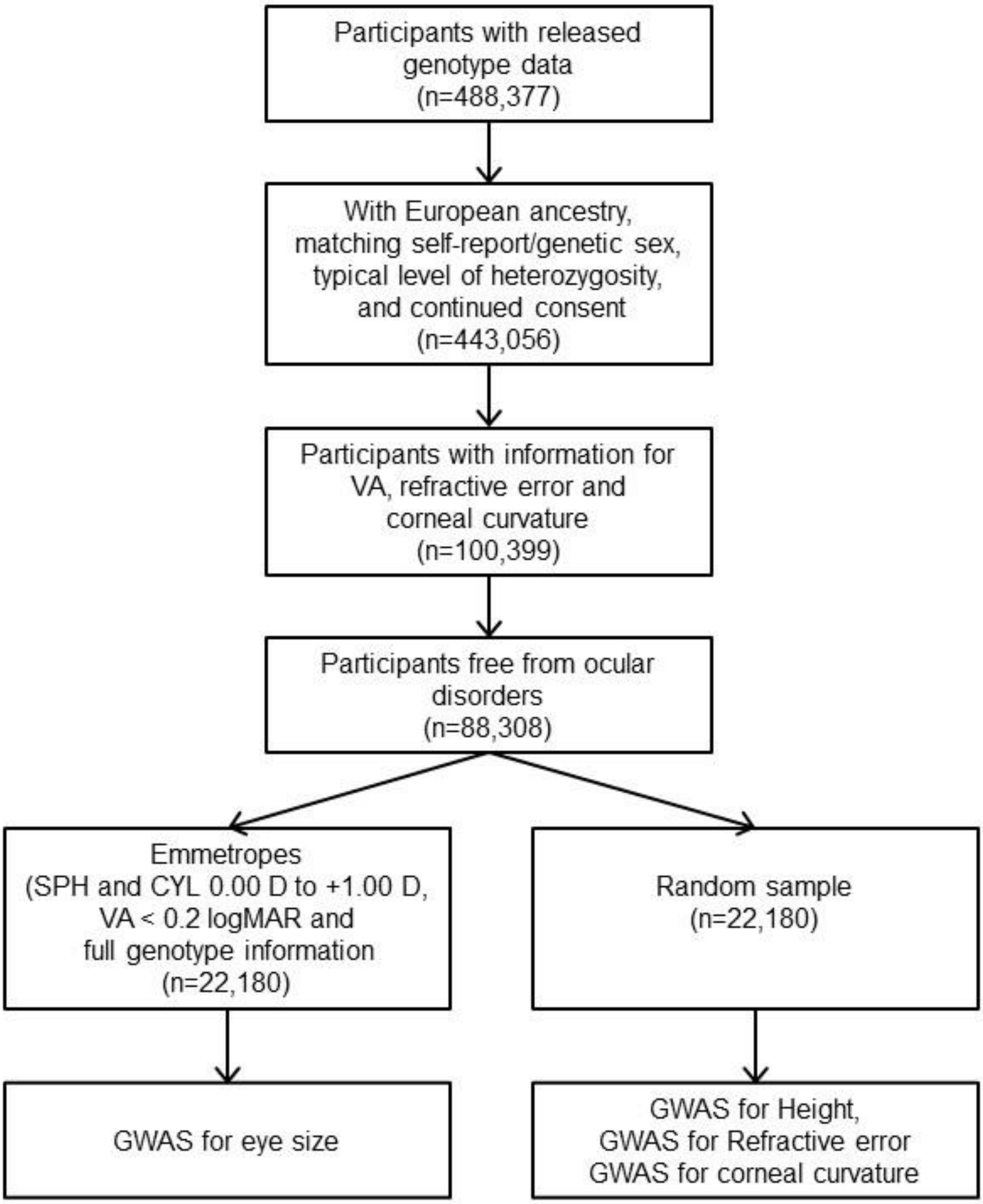

Figure S2. Replication of eye size-associated variants with traits in ALSPAC participants. The 32 genetic variants associated with eye size (corneal curvature in emmetropes from UK Biobank) were tested for association with refractive error, corneal curvature, axial length, and height, in a sample of n=1,848 participants aged 15 years-old. Blue squares indicate association at P<0.10 (panel A) and matched direction of association with the original GWAS (panel B).

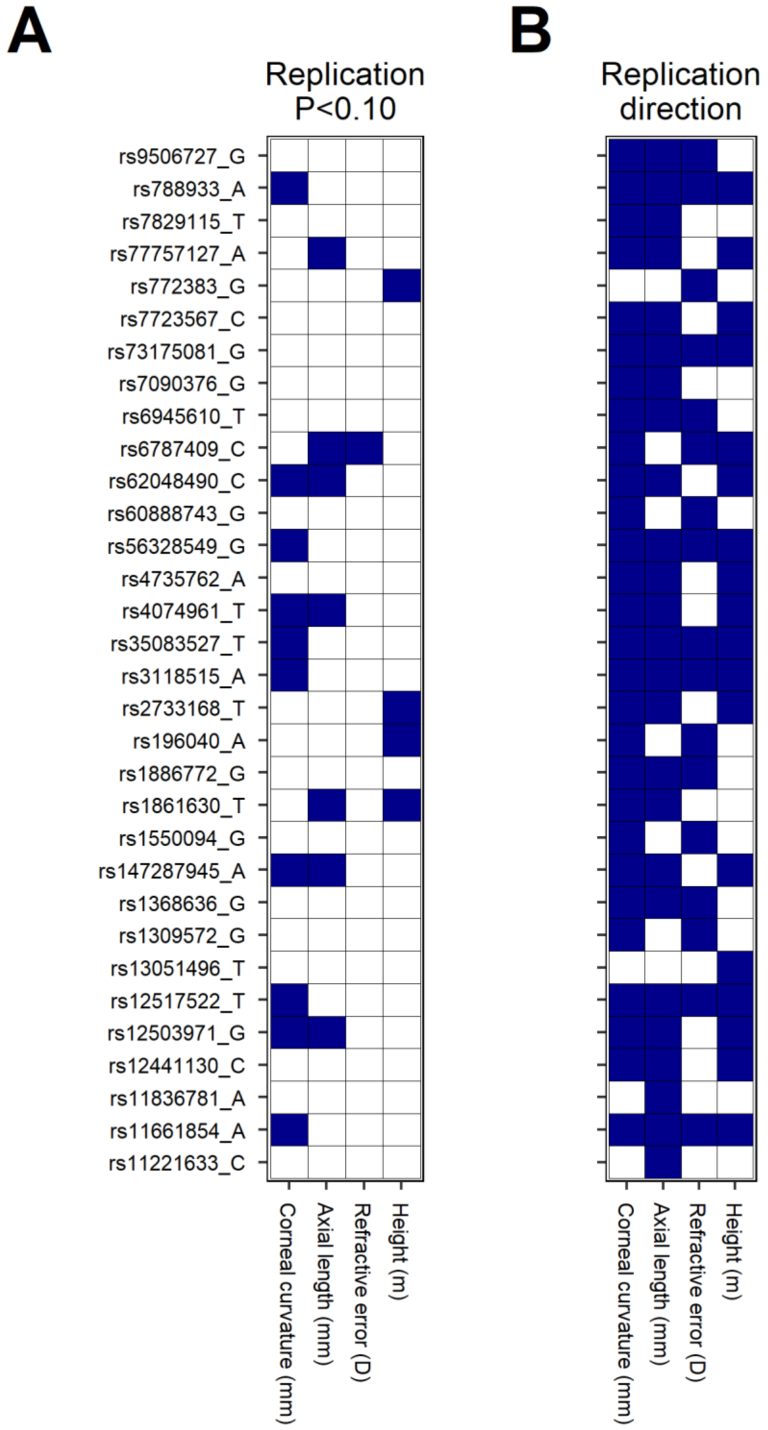

Figure S3(a). Sensitivity analyses to test robustness of the genetic correlation results. For these analyses, an alternative definition of emmetropization was used. Namely, emmetropic eyes were defined as those with a mean spherical equivalent (MSE) refractive error of -0.50 ≤ MSE ≤ +0.50 D and with a VA <0.2 logMAR. There were a total of 27,569 UK Biobank individuals with at least 1 emmetropic eye who met this criterion and who were included in a GWAS for corneal curvature. Genetic correlations were calculated as in the main analysis.

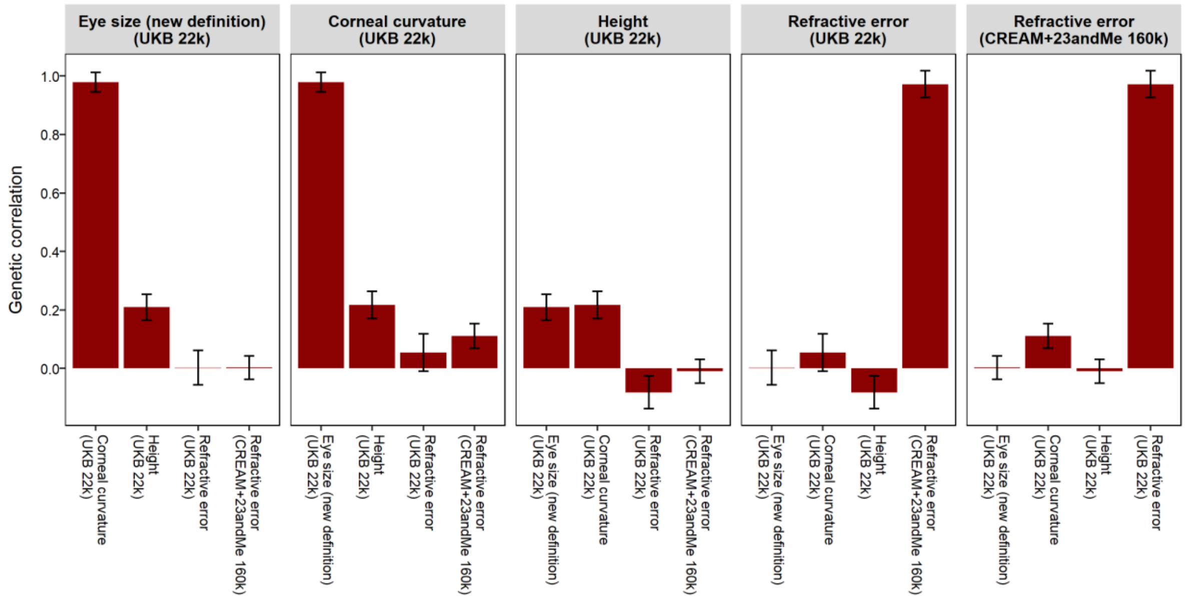

Figure S3(b). Sensitivity analyses to test robustness of the genetic correlation results. For these analyses, an alternative definition of emmetropization was used. Namely, emmetropic eyes were defined as those with a mean spherical equivalent (MSE) refractive error of -0.50 ≤ MSE ≤ +0.50 D and with a VA <0.2 logMAR (the same definition used in Figure S3a). However, here, only the 12,028 UK Biobank individuals who were classified as emmetropic in ***both*** eyes were included in the GWAS for corneal curvature. Genetic correlations were calculated as in the main analysis.

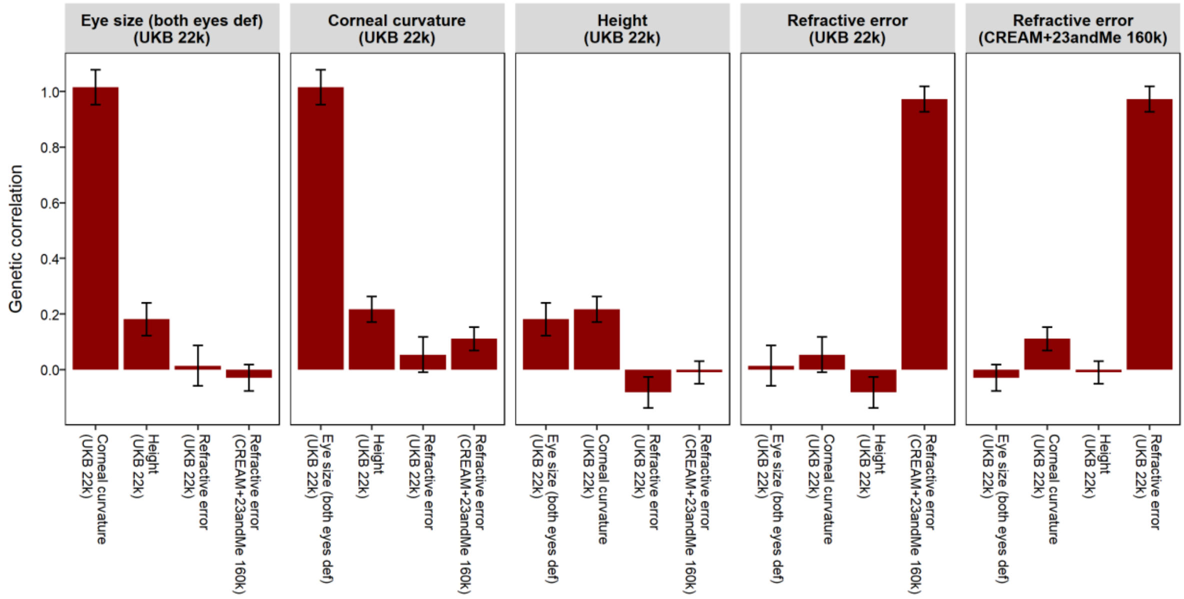

Figure S3(c). Sensitivity analyses to test robustness of the genetic correlation results: Inclusion of height as a covariate in the GWAS analysis for corneal curvature in emmetropes. Emmetropic eyes were defined as in the main analysis. There were a total of 22,180 UK Biobank individuals who were classified as emmetropic in at least 1 eye and who were included in a GWAS for corneal curvature. Height was included as a covariate in the GWAS analysis, along with the covariates used in the main analysis. Genetic correlations were calculated as in the main analysis.

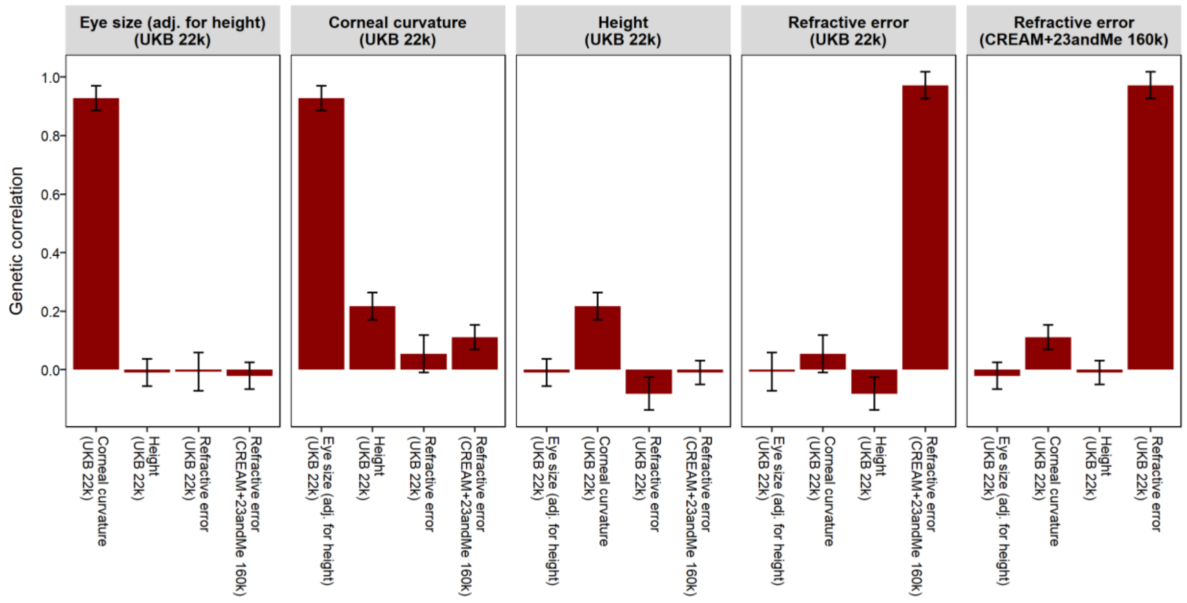

Supplement: Supplement 1 [file iovs-62-13-24_s001.pdf]
